# Supplementary material for: In Vitro Susceptibility of Cryptosporidium parvum to Plant Antiparasitic Compounds
Source: Pathogens. 2022 Dec 30;12(1):61. doi: 10.3390/pathogens12010061 (PMC9863366; doi:10.3390/pathogens12010061)
Supplement: Supplementary file 1 [file pathogens-12-00061-s001.zip › Supplementary Material S1.pdf]

Supplementary Material S1

Confirmation of *Cryptosporidium parvum* infectivity

Methods:

The immunofluorescence reagent EasyStain™ (BioPoint Pty. Ltd, Australia), which contains 4,6' diamino-2-phenylindole dihydrochloride (DAPI), was used to determine the presence of four nuclei (sporozoites) within the *C. parvum* oocysts. The stained samples were visualised using a fluorescence and differential interference contrast (DIC) microscope (Olympus BX51, Olympus America Inc., Lake Success, New York) with appropriate fluorescence filters.

Results:

The microscopic observations confirmed the successful *C. parvum* invasions in HCT-8 cell monolayers. The size, shape, and morphology of *C. parvum* oocysts stained with EasyStain™ used in the present study agreed with the *C. parvum* specific characteristics (Figure S1).

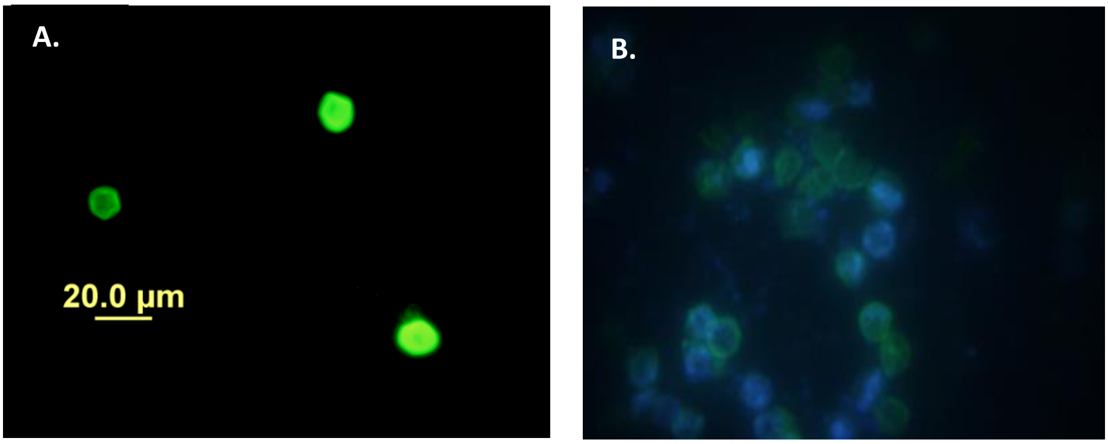

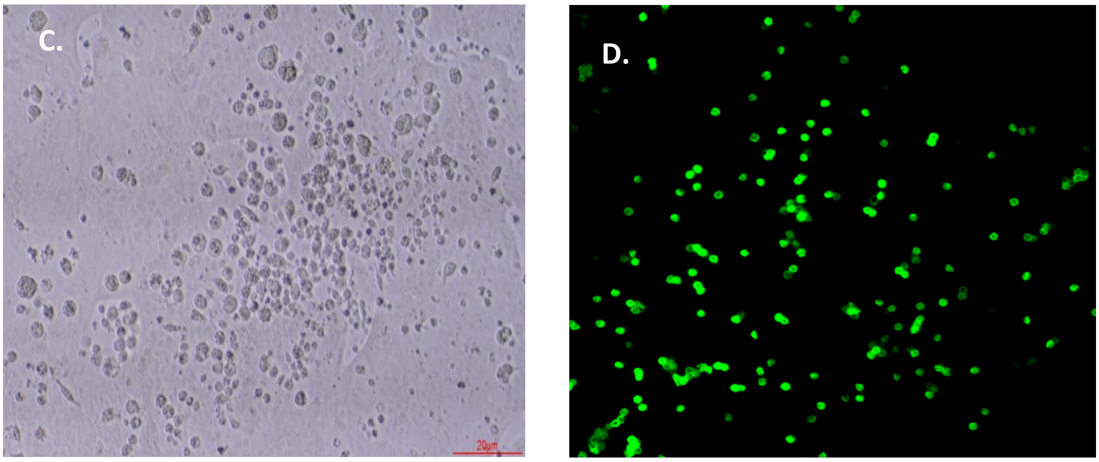

Figure S1. Micrograph of **A.** *C. parvum* oocysts stained with EasyStain™ without DAPI **B.** *C. parvum* oocysts stained with EasyStain™ with DAPI **C.** *C. parvum* infected HCT-8 cell monolayers **D.** *C. parvum* infected HCT-8 monolayers stained with EasyStain™
