# Supplementary material for: In Vitro Susceptibility of Cryptosporidium parvum to Plant Antiparasitic Compounds
Source: Pathogens. 2022 Dec 30;12(1):61. doi: 10.3390/pathogens12010061 (PMC9863366; doi:10.3390/pathogens12010061)
Supplement: Supplementary file 1 [file pathogens-12-00061-s001.zip › Supplementary Material S2 Revised -2048930.pdf]

**Supplementary Material S2**

## Immunofluorescence assay

***Methods:***

The immunofluorescence methods with specific markers are considered to provide easy visualisation of *Cryptosporidium* (Schets et al., 2005; Boxell et al., 2008; Hijjawi, 2010). The fluorescent polyclonal antibody Sporo-Glo™ has proven to be useful in detecting *Cryptosporidium* infection foci in host cell monolayers and identifying other life cycle stages (Boxell et al., 2008). Sporo-Glo™ enables the visualisation of both extracellular (sporozoites and merozoites) and intracellular (trophozoites, meronts and gamonts) developmental stages of *Cryptosporidium* (Boxell et al., 2008).

An immunofluorescence assay was performed to evaluate the *C. parvum* infection intensity using a fluorescein-conjugated specific polyclonal antibody (Sporo-Glo™, Waterborne Inc., New Orleans, LA, USA) according to the manufacturer's protocol. Briefly, the existing medium was aspirated from the wells, and the cell monolayers were fixed with 100% (vol/vol) methanol (Fisher Scientific, UK) for 10 min at room temperature (RT). Methanol was then aspirated off from the wells, and 500 µL of blocking buffer was added to each well and incubated for 30 min at RT. The buffer was removed from the wells, and 200 µL of 1x Sporo-Glo™ antibody reagent [1/100 (vol/vol) (diluted in PBS)] was added to each well. The light-protected plates were then incubated at RT with shaking for 1 h. Antibody reagent was drained out from the wells, and the wells were rinsed with 500 µL of PBS for 4 times. Each well was then mounted with one drop of mounting medium. Different *in vitro* *Cryptosporidium* intracellular life stages were counted in random microscopic fields ($n$ = 6) under an Olympus BX51 microscope (Olympus America Inc., Lake Success, New York). Three

replication wells of a 48-well plate were used for each concentration. The mean number of intracellular forms from 3 wells (*n* = 3) was used to indicate infection intensity. Wells containing plant compounds were compared to infected and untreated control wells, which were considered to have 100% parasite development.

***Results:***

The immunofluorescence assay was used to assess the *in vitro* activity of test compounds against *C. parvum* growth. The fluorescence-microscopic observation revealed the presence of intracellular stages in the infected and treated monolayers and the untreated control wells. Compared to the untreated controls, a higher inhibition of *C. parvum* growth was observed with *Curcuma longa*, *Piper nigrum*, *Embelia ribes*, and *Nigella sativa* (Fig. S2).

In the current study, the Sporo-Glo™ stained *C. parvum* infected and treated monolayers were compared to that of untreated control wells, and the findings were compatible with the qPCR results.

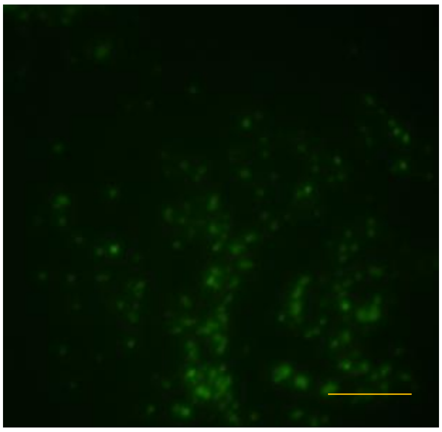

**Untreated control**

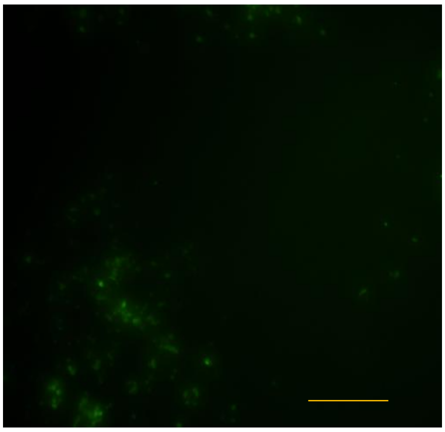

***Curcuma longa* at 0.5 µg/mL**

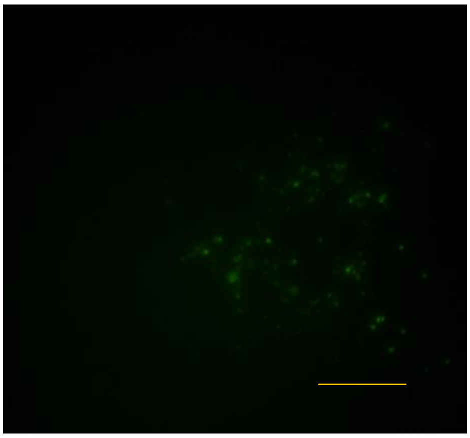

***Embelia ribes* at 0.5 µg/mL**

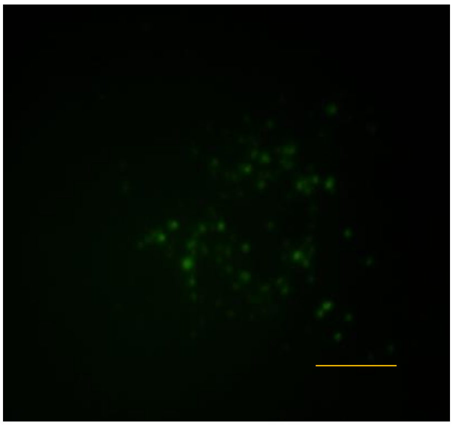

***Piper nigrum* at 0.5 µg/mL**

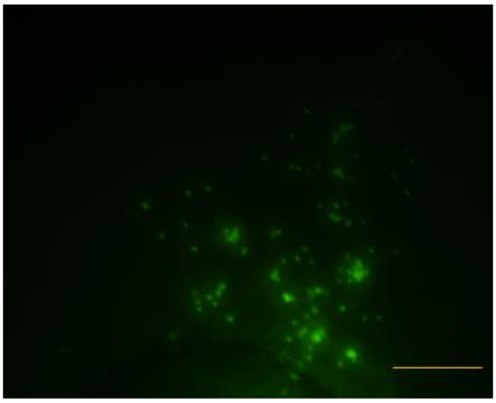

***Nigella sativum* at 0.5 µg/mL**

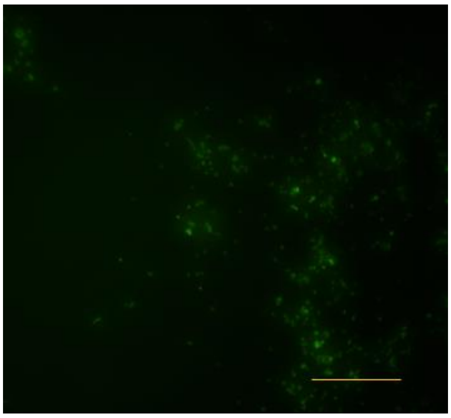

**Trifluralin at 10 µg/mL**

**Figure S2.** Representative micrographs of HCT-8 cell monolayers infected with *C. parvum* oocysts, treated with test compounds, and stained with Sporo-Glo™, a polyclonal antibody, specific for a *Cryptosporidium* antigen expressed in sporozoites and intracellular stages
