# Supplementary material for: In Vitro Susceptibility of Cryptosporidium parvum to Plant Antiparasitic Compounds
Source: Pathogens. 2022 Dec 30;12(1):61. doi: 10.3390/pathogens12010061 (PMC9863366; doi:10.3390/pathogens12010061)
Supplement: Supplementary file 1 [file pathogens-12-00061-s001.zip › Table S1.pdf]

**Table S1. Mean % Cell viability of HCT-8 cells exposed to plant extracts**

| | Mean % Cell Viability (± SEM) | | | | | |
|---|---|---|---|---|---|---|
| | Concentration (µg/mL) | | | | | |
| | **2.05** | **6.16** | **18.5** | **55.5** | **166** | **500** |
| *Allium sativum* L. (garlic) | 97.34 ± 1.05 | 95.79 ± 0.19 | 95.11 ± 0.19 | 96.13 ± 0.53 | 96.13 ± 0.77 | 98.48 ± 0.90 |
| *Boswellia serrata* Roxb. (Indian frankincense) | 97.13 ± 1.44 | 98.25 ± 0.91 | 98.38 ± 1.05 | 98.17 ± 1.54 | 96.73 ± 1.39 | 97.34 ± 1.04 |
| *Centella asiatica* (L.) urban (Gotu kola) | 99.78 ± 0.27 | 98.51 ± 0.86 | 96.09 ± 0.80 | 98.75 ± 1.25 | 98.10 ± 1.14 | 98.98 ± 0.60 |
| *Cucurbita pepo* L. (pumpkin) | 88.01 ± 1.91 | 90.91 ± 1.40 | 91.83 ± 1.18 | 86.79 ± 0.81 | 93.15 ± 1.04 | 88.14 ± 0.58 |
| *Curcuma longa* L. (turmeric) | 97.14 ± 1.46 | 92.71 ± 0.67 | 96.26 ± 1.40 | 93.17 ± 0.73 | 97.08 ± 1.20 | 97.47 ± 0.88 |
| *Embelia ribes* Burm. f. (false black pepper) | 97.07 ± 0.34 | 96.07 ± 2.19 | 93.71 ±1.00 | 93.04 ± 1.18 | 95.68 ± 2.63 | 94.05 ± 0.80 |
| *Glycyrrhiza glabra* L. (liquorice) | 87.37 ± 1.04 | 89.31 ± 1.40 | 89.75 ± 1.26 | 93.69 ± 1.65 | 93.30 ± 1.09 | 91.77 ± 0.47 |

| | | | | | | |
|---|---|---|---|---|---|---|
| *Moringa oleifera* Lam. (drumstick) | 89.39 ± 1.13 | 85.21 ± 0.54 | 89.13 ± 1.50 | 86.06 ± 1.01 | 89.87 ± 1.58 | 92.14 ± 0.40 |
| *Nigella Sativa* L. (black cumin) | 98.56 ± 1.44 | 99.53 ± 0.47 | 96.69 ± 1.72 | 94.97 ± 2.52 | 93.46 ± 2.43 | 98.13 ± 1.87 |
| *Piper nigrum* L. (black pepper) | 99.04 ± 0.51 | 96.05 ± 1.98 | 95.79 ± 2.26 | 97.28 ± 0.56 | 99.28 ± 0.72 | 98.79 ± 1.21 |
| *Thymus vulgaris* L. (thyme) | 98.83 ± 0.23 | 96.98 ± 0.97 | 96.56 ± 1.79 | 98.77 ± 1.23 | 99.40 ± 0.60 | 98.62 ± 0.81 |
| *Tribulus terrestris* L. (goat's-head/ caltrop) | 97.72 ± 1.18 | 96.26 ± 1.49 | 99.03 ± 0.97 | 98.88 ± 1.12 | 94.87 ± 2.62 | 99.57 ± 0.43 |
| *Vitex negundo* L. (Chinese chaste tree) | 97.55 ± 1.92 | 99.39 ± 0.61 | 97.24 ± 1.43 | 98.35 ± 1.07 | 99.18 ± 0.82 | 96.49 ± 1.80 |
| Trifluralin | 84.18 ± 2.98 | 84.24 ± 2.50 | 84.16 ± 1.68 | 85.31 ± 1.77 | 86.74 ± 1.31 | 86.21 ± 2.98 |
